# Relations between phonological production, grammar and the lexicon in bilingual French-English children

**DOI:** 10.1177/13670069211031987

**Published:** 2021-07-20

**Authors:** Margaret Kehoe, Margaret Friend, Diane Poulin-Dubois

**Affiliations:** University of Geneva, Switzerland; San Diego State University, USA; Concordia University, Canada

**Keywords:** Phonology, vocabulary, grammar, phonological development, lexical development, grammatical development

## Abstract

**Aims and objectives:**

This study examines multiple associations between language domains in bilingual children with a focus on phonology. Previous studies indicate within- but not cross-language associations between vocabulary and grammar in bilingual children. We investigate whether the relation between phonology and other language domains differs from the one reported between vocabulary and grammar.

**Methodology:**

Canadian French-English bilingual children (*n* = 31), aged 31 months, participated in 2 free-play sessions, from which lexical, grammatical and phonological information was extracted. The children’s parents completed the MacArthur-Bates Communicative Developmental Inventories and its Canadian French adaptation providing additional information on vocabulary and grammar in each of the children’s languages. They also completed a questionnaire on their children’s exposure to French and English.

**Data analysis:**

Within and cross-language relations between phonology, vocabulary and grammar were investigated using correlational analyses and mixed logistic regression.

**Findings:**

Correlational analyses did not reveal significant cross-language relations between phonology, vocabulary and grammar. However, mixed logistic regression, which controlled for language exposure effects, indicated that phonology was influenced by vocabulary and grammar both within and across languages.

**Originality:**

This study is one of the first to study cross-domain relations involving phonology in young bilingual children.

**Implications:**

Overall, the findings suggest that phonology displays a pattern of relations that is different from other language domains engendering between-language effects due to a language-general component.

## Introduction

Researchers in child language development have long recognized the importance of studying the relation between different language domains as well as focusing on a single domain (Stoel-Gammon, 2011). Robust relations between vocabulary and grammar (Braginsky et al., 2015; Dale et al., 2000; Thal et al., 1997), vocabulary and phonology (Kehoe et al., 2015; Petinou & Okalidou, 2006; Rescorla & Ratner, 1996; Smith et al., 2006) and grammar and phonology (Bortolini & Leonard, 2000; Gerken, 1996; Lleó & Demuth, 1999) have been documented in monolingual children. Studying cross-domain relations is also of interest in bilingual children because there is the potential to study multiple connections, within- and between-language, while holding the child (i.e. general language) factor constant (Marchman et al., 2004; Pearson et al., 1997). The link between vocabulary and grammar (i.e. morphosyntax) in bilingual children has already been the subject of some attention (Conboy & Thal, 2006; Marchman et al., 2004). Studies show strong within- but weak between-language associations. That is, vocabulary in one language influences grammar in the same but not in the other language.

The present study investigates cross-domain associations between phonology, vocabulary and grammar in bilingual children. We examine whether the relation between phonology and other language domains patterns differently from the one between vocabulary and grammar. A collection of studies report correlations between the phonological scores of the two languages (Cooperson et al., 2013; Keffala et al., 2020; Montanari et al., 2018; Scarpino, 2011; Scarpino et al., 2019) and some studies report between-language correlations in the phonology-grammar and phonology-vocabulary relations: phonology is related to the grammar and vocabulary of both languages (Cooperson et al., 2013; Kehoe & Havy, 2019). We hypothesize that the dependence of children’s phonological skills upon a language-general component may lead to greater between-language associations than is observed between vocabulary and grammar (Kehoe, 2011, 2015; Montanari et al., 2018). We aim to test this hypothesis by studying cross-domain associations in French-English bilingual children, aged 31 months, thus extending previous studies focused largely on older children and/or on Spanish-English bilinguals (Cooperson et al., 2013; Keffala et al., 2020; Scarpino et al., 2019). In the following sections, we define within- and between-language associations, summarize studies which have examined within- and between-language associations in bilingual children, and clarify why we consider the association between phonology and other domains to be different from the one between vocabulary and grammar.

## Within- and between-language associations between language domains in bilingual children

We consider within-language associations to reflect facilitative effects of one language skill on another within the same language and between- or cross-language associations to reflect the facilitative effect of a language skill in one language on a language skill in the other. An example of a between-language effect would be good phonological skills in English being associated with a large vocabulary size in French. Between-language relations may also be non-facilitative as reflected in negative correlations between two language domains (e.g. good phonology skills in English being associated with a small vocabulary size in French). These ‘subtractive’ effects may reflect either the influence of relative language exposure or of environments in which the first language (L1) is not supported (Hoff et al., 2018). However, the present study aims to document facilitative between-language effects.

### Vocabulary and grammar

Marchman et al. (2004) were among the first to explore within- and between-language effects in lexical and grammatical relations in bilingual children. They hypothesized that between-language associations should be observed if general language learning (i.e. cognitive skills and environmental influences) underlies lexical-grammatical relations. In contrast, within-language associations would suggest that lexical-grammatical relations are language-specific. They conducted two studies on bilingual Spanish-English children, aged 24 and 27 months. The first study compared expressive vocabulary size to grammatical ability, using parent report in each language, in a large sample of bilinguals (*n* = 113). Measures of grammatical ability included: the mean length of the three longest utterances (ML3) and a complexity score, in which reporters selected from 37 pairs of phrases (one phrase containing grammatical markers and one not) the phrase which most characterized their children’s speech. In the second study, with a smaller sample (*n* = 26), they extracted vocabulary and grammar measures, number of different words (NDW) and mean length of utterances-in words (MLU), from spontaneous language samples. The results were almost identical across the two studies: within-language were stronger than between-language associations, providing support for language-specific over language-general accounts of language learning.

Conboy and Thal (2006) obtained similar results in longitudinal research with Spanish-English bilingual children aged 20 to 30 months. Like Marchman et al. (2004), they obtained parent reports on vocabulary and grammatical measures. In addition, they calculated conceptual vocabulary (i.e. the number of different concepts that a child knows), positing that bilingual children might pool linguistic concepts across languages to extract grammatical rules. Hierarchical growth curve models indicated that language-specific vocabulary predicted grammatical development in the use of predicates and closed class items, whereas conceptual vocabulary did not contribute additional variance.

More recent studies examining lexical and grammatical associations in both simultaneous and sequential bilingual children confirm the earlier findings. They have all found evidence of strong within- and few if any cross-language connections (Hoff et al., 2018; Kohnert et al., 2010; Pham, 2016; Simon-Cereijido & Gutiérrez-Clellen, 2009; Simon-Cereijido & Méndez, 2018, 2020). Nevertheless, some of these studies, including Marchman et al. (2004) and Conboy and Thal (2006), have reported isolated cross-language effects. Marchman et al. (2004) noted that Spanish vocabulary accounted for a small amount of unique variance in English grammar once English vocabulary was accounted for. Similarly, Conboy and Thal (2006) found a positive relation between the number of English words produced by children at a given time point (i.e. 28–31 months) and their Spanish ML3. A handful of studies have also found evidence of between-language effects in vocabulary measures (Dixon, 2011; Kohnert et al., 2010; Pham, 2016). Dixon (2011), for example, found that the English vocabulary of bilingual kindergarten children growing up in Singapore was also predicted by their mother-tongue vocabulary and Kohnert et al. (2010) found a modest positive correlation between the NDW produced in the L1 and second language (L2) of sequential Hmong-English bilinguals. Despite these isolated reports, the overall evidence for cross-language associations between lexical and grammatical domains is limited.

### Phonology and other language domains

In contrast to the findings in vocabulary and grammar, there are robust findings showing that phonological skills in one language predict phonological skills in the bilingual’s other language. Keffala et al. (2020) examined factors which predict phonological abilities in 695 Spanish-English bilinguals, aged 3 to 6 years. They found that cross-linguistic phonological skills had the largest effect on consonant accuracy in each language. That is, good phonological skills in one language were associated with good phonological skills in the other language, and vice versa. Similar findings were reported by: Scarpino et al. (2019) with 199 Spanish-English bilinguals, aged 3 to 6 years; Cooperson et al. (2013) with 186 Spanish-English bilinguals having a mean age of 5 years 9 months; and Montanari et al. (2018) with 35 Spanish-English bilinguals tested at 2 age points 3;7 and 4;7 years.

There are also robust findings showing that vocabulary and grammar skills in one language predict phonological skills in the same language. Meziane and MacLeod (2017) reported significant correlations between expressive (but not receptive) vocabulary and percent consonants correct (PCC) scores in French second-language learners, aged approximately six years. Similarly, Kehoe and Giradier (2020) found significant correlations between French expressive vocabulary and French phonological measures (e.g. PCC, percent codas and clusters correct) in French simultaneous bilinguals, aged three to six years. In addition, two recent large-scale studies of Spanish-English bilinguals, aged three to six years, report that expressive vocabulary ability predicts consonant accuracy and phonological whole-word proximity in the same language (Keffala et al., 2020; Scarpino et al., 2019). In a similar vein, studies have documented significant correlations between phonology and morphosyntax, as measured by MLU, on a language-specific basis in bilingual children (Goldstein et al., 2010; Montanari et al., 2018).

Given the presence of between-language effects in the phonology of the two languages, and within-language effects between phonology, vocabulary and grammar, a corollary would be to find evidence of between-language cross-domain effects. To date, few authors have examined between-language relations implicating phonology and other language domains, but some studies exist, namely those of Cooperson et al. (2013) and Kehoe and Havy (2019). Cooperson et al. (2013) investigated within- and between-language relations in phonology, semantics and grammar. Spanish-English bilingual children (*n* = 186) with a mean age of 5;9 were administered the bilingual English-Spanish assessment (BESA) (Peña et al., 2018)^1^ which included phonological, semantic and morphosyntactic components. The phonological measure was a picture naming task which assessed the number of consonant and vowel targets correctly produced. The semantic measure tested both receptive and expressive vocabulary and children’s knowledge of word associations and lexical categories. The morphosyntactic measure consisted of a cloze task, which tested knowledge of grammatical structures, and a sentence repetition task. Finally, narrative language samples were collected from which three measures were extracted: NDW, MLU and percent grammatical utterances. Cooperson et al. (2013) reported stronger within- than between-language correlations when examining phonology, grammar and semantics in bilingual children; nevertheless, in regression models, percent English grammatical utterances explained unique variance in Spanish phonology, and Spanish morphosyntax explained unique variance in English phonology suggesting a between-language component to the phonology–grammar relation as well. Cooperson et al. (2013) posited that language-general phonological skills provide a foundation for the acquisition of grammar in both languages of the bilingual.

Kehoe and Havy (2019) included both French and total vocabulary as predictors of French phonological development in a study of French-speaking bilingual children, aged 30 months. Total vocabulary was a significant predictor of some phonological measures (i.e. PCC, word final consonant accuracy), whereas French vocabulary was not significant in any model. The fact that total, rather than language-specific, vocabulary was more closely correlated with phonology in French provides some evidence that phonology is influenced by cross-language measures. However, in order to fully examine this issue, phonological production and vocabulary measures in both languages are needed, which is one of the goals of the current study; namely, to examine within- and between-language associations in phonology, vocabulary and also grammar.

### Why is phonology different?

Why should the association between phonology and other domains be different from that of vocabulary and grammar? We conceptualize phonological development as comprising: (a) a biologically based component related to the development of speech-motor and articulatory skills; and (b) a cognitive-linguistic component related to acquiring the phonological system of the ambient language (Stoel-Gammon, 2011). The speech motor and articulatory skills underlying phonology may be constant resulting in strong similarities between the two phonological systems of the bilingual. For example, studies on bilinguals with motor speech impairment (e.g. childhood apraxia of speech) show similar patterns across languages on motor-based tasks suggesting that aspects of motor control are language-neutral (Preston & Seki, 2011). Whereas the cognitive-linguistic (phonological) component should be language-specific, it may lead to ‘language-general-like’ effects due to the many shared segmental and phonotactic structures across languages (Keffala et al., 2020; Parra et al., 2011; Scarpino et al., 2019).

Our proposal that phonology acts differently from vocabulary or morphosyntax is consistent with the notion of bilingual profile effects (Oller et al., 2007). Monolingual-bilingual differences are more extreme in certain language domains than others because of the distributed nature of bilingual knowledge. Distributed knowledge is particularly evident in vocabulary acquisition whereby the form-meaning relation is essentially arbitrary and must be learned on a language-specific basis. It is not necessarily evident in phonics (knowing letters ‘p’, ‘b’ and ‘s’) due to the strong commonalities in letter to sound correspondence across languages. We posit that phonological production may operate similarly to phonics. Indeed, studies attest to strong differences between typically developing monolingual and bilingual children in the areas of lexical and morpho-syntactic development (Hoff et al., 2012) and fewer differences in the area of speech sound development (Hambly et al., 2013).^2^

In line with this proposal is some evidence, based on the literature, that the influence of language experience is stronger for vocabulary and morphosyntax than for phonology. Language experience is often measured using parent-reported estimates of frequency of language input and output, language proficiency tests or parental rating scales. Regardless of how it is measured, there is a large literature showing that it is highly correlated with bilingual children’s vocabulary and morphosyntactic performance (Hoff et al., 2012; Legacy et al., 2016; Parra et al., 2011; Pearson et al., 1997; Thordardottir, 2011, 2015; Unsworth, 2016). For example, Hoff et al. (2012) found moderate to high significant correlations between percent English home language use and parent-reported vocabulary and grammar in bilingual Spanish-English children, aged 22 to 30 months. Similarly, Legacy et al. (2016) report significant correlations between language exposure and vocabulary size in French-English bilingual children followed longitudinally from 18 through to 31 months. Some of the children from their study are tested in the current one.

In the area of phonology, the influence of language experience tends to be less strong. Some authors have found it to be a significant predictor of phonological accuracy (Morrow et al., 2014; Ruiz-Felter et al., 2016), whereas others have reported it to have only modest effects on phonology (Almeida et al., 2012; Cooperson et al., 2013; Goldstein et al., 2005, 2010). We hypothesize that even if a child has reduced language experience in one language, bootstrapping from the phonology of the other language may compensate for the reduced language experience. Thus, phonology may be less susceptible to the influence of language experience than other language domains, consistent with our expectation that a stronger language-general component is implicated in the phonological systems of bilinguals, as compared to their lexical or grammatical systems. As a way of testing this, we examine the influence of language exposure on performance across these different language domains.

## Summary and current study

In sum, studies which have examined interactions between different domains of language in bilingual children find strong support for within-language correlations. Studies provide less support for between-language relations; however, some exceptions have been found, particularly in the case of phonology, suggesting that a language-general component underlies associations between phonology and other language domains (Cooperson et al., 2013; Keffala et al., 2020). The bulk of the studies examining between-language correlations in phonology have focused on older children, whereas this study examines relations between language domains in younger children selecting an age-range similar to that of Marchman et al. (2004) and Conboy and Thal (2006) in their studies of vocabulary-grammar connections.

We explore relations between phonology, vocabulary and grammar in bilingual French-English children, aged 31 months, and examine the relative effects of language exposure on the different language domains. The children took part in free-play sessions, from which vocabulary, grammatical and phonological information was extracted. Parents completed the Canadian French and American English versions of the MacArthur-Bates Communicative Developmental Inventories (MCDI), providing us with additional information on the children’s vocabulary and grammatical ability in both of their languages. They also completed a questionnaire on language exposure which was used to determine if the children were English or French dominant.

First, we examine correlations between proportion language exposure and phonological, vocabulary and grammatical measures in each language. We also examine correlations between language measures within and across languages. We predict that language exposure may influence phonology to a lesser extent than it influences vocabulary and grammar, and that there will be not only significant within- but also cross-language correlations between phonology and other language measures. Second, using mixed logistic regression, we examine whether language measures influence phonological performance in English and French. We predict that phonological performance in one language will be influenced by the vocabulary and/or grammatical abilities of the same language. In addition, we predict that phonological performance in one language will be influenced by the phonological, vocabulary, and/or grammatical abilities of the other language.

## Method

This study is part of a larger project whose main purpose was to examine the association between early language comprehension and later literacy development. The larger study involved 58 bilingual French-English children tested longitudinally from 16 through to 60 months. We focus on a sub-sample of these children at a single age-range, approximately 30 months.

## Participants

Forty-two French-English bilingual children were selected from the larger database. We excluded children who obtained very low vocabulary scores in both of their languages (i.e. below the 10th percentile) based on their MCDI result in French and English. The criterion for being bilingual was that they were exposed at least 20% of the time to a second language; however, one child, who had only 18% exposure to French, was still included because he did not differ qualitatively from the other children. Despite this criterion, eight children had to be eliminated because they did not produce sufficient numbers of phrases in one of their target languages during the free-play sessions. In addition, three children were eliminated due to missing audio tapes and transcriptions. This left a complete data set of 31 bilingual children (10 girls) who had a mean age of 31 months (range: 28–33 months). All children had been exposed to French and English from birth. Among the children, there were 6 trilinguals who were minimally exposed to a third language (mean proportion of exposure = .14). Most children were first-born (*n* = 23); the remaining children were second- (*n* = 5) and third-born (*n* = 3). All children were typically developing with normal hearing and vision. A summary of the demographic characteristics of the participants is provided in Table 1.

**Table 1.** Demographic characteristics of participants.

	Mean (*SD*)	Range
Age (months)	30.71 (1.01)	28–33
Proportion English language exposure	.45 (.15)	.25–.83
Proportion French language exposure	.53 (.16)	.18–.77
Maternal education (years)	15.94 (1.88)	11–19
Paternal education (years)	15.57 (2.61)	8–19
Income (Canadian dollars)	113,724 (73,049)	18,000–300,000

## General procedure

Children attended 2 free-play sessions of 20 minutes duration (1 in French and 1 in English) in the Cognitive and Language Development Laboratory of Concordia University in Montréal. The visits were scheduled one week apart. The language of testing was counterbalanced across participants. The children were accompanied by their caregiver, who in most cases was a bilingual French-English parent.

## Materials

### Language Exposure Assessment Tool

Language exposure was estimated by using the Language Exposure Assessment Tool (LEAT), which is an excel-based parent interview (DeAnda et al., 2016; https://pubs.asha.org/doi/suppl/10.1044/2016_JSLHR-L-15-0234) administered over the phone prior to the child’s visit. The LEAT obtains information on the languages spoken by the interlocutors who interact regularly with the child, whether the interlocutors are native speakers, and the number of hours of talking or being overheard by the child in each language. The program yields estimates of the relative exposure to each language in hours per day, hours per week, and proportion exposure. We used proportion exposure to each language as the estimate of relative exposure. Children who received greater exposure in English were designated as English dominant (*n* = 11) and children who received greater exposure to French were French dominant (*n* = 20).

### MCDI and L’Inventaire MacArthur de Développement de la Communication

The American English MCDI: Words and Sentences (Fenson et al., 2007) and its Canadian French adaptation, L’Inventaire MacArthur de Développement de la Communication (IMDC): Mots et Phrases (Trudeau et al., 1999) were given to the parents at the time of testing. The MCDI contains a parent-report checklist of 680 words on which caregivers indicate the words their children say. The MCDI has high internal consistency (Cronbach’s α = .96), and strong test-retest reliability. The IMDC (referred to as the French MCDI) was normed on children acquiring Québécois French and has strong test-retest reliability. It contains 688 words.

Expressive vocabulary in each language was estimated as the number of words parents report that children produce. In addition, parents listed 3 of the longest sentences their child had produced recently and marked the sentence that best characterized the way their child talked (from a set of 37 pairs; e.g. *Daddy car* vs *Daddy’s car*). In the former, we determined the mean number of words in the three longest sentences (ML3); in the latter, we calculated the number of sentences which contained grammatical markers (GramCom). One parent did not complete information on their child’s grammatical abilities in both languages. Another parent did not provide examples of the three longest phrases in French.

### Free-play spontaneous language sample

Children interacted with their parents in one free-play session in each language. Many parents were French-English bilinguals and, in over half the cases, the same parent was present for both the English and French sessions. Parents were told to play as they would at home and to speak to their child in either English or French depending on the target language for the session. During the free-play session, dyads played with a complex toy, either a farm or a house. Children played with a different toy at each visit to control for repetition effects. The toy set used for each language was counterbalanced across participants. The language samples were recorded using a portable digital tape-recorder (Marantz PMD620).

### Data-coding and analyses

#### Semantic and grammatical analysis

Language samples were transcribed using the Systematic Analysis of Language Transcripts software (SALT; Miller & Iglesias, 2012). Eight transcribers completed three to eight transcriptions each. Transcribers were fluent in English and French. Prior to starting work on the study, transcribers completed online training provided by the SALT Software, LLC. They performed practice transcriptions and were required to meet a minimum inter-rater agreement of .8. A research assistant performed reliability transcription/coding for approximately 15% of the transcripts. Word-level agreement was .90 for the English and .89 for the French transcripts.

Using the SALT software, MLU calculated in words and NDW were automatically generated for each child. Many children displayed code-switching, defined as the presence of non-target words and phrases (i.e. the presence of English words and phrases in a French free-play session). MLU and NDW were calculated only for non-code-switched words and phrases.^3^ The average number of complete and intelligible French utterances used to determine NDW and MLU was 105 (*SD* = 40), and the average number of English utterances was 127 (*SD* = 60).

#### Phonological analysis

We employed PCC as the phonological production measure. It was obtained using Phon, a software program designed for the analysis of phonological data (Rose et al., 2006). The digitized recordings of the language samples were segmented into utterances, glossed and phonetically transcribed. Three French-speaking undergraduate students with experience in phonetic transcription performed the analyses in French.^4^ A native English speaker trained in phonetic transcription performed the analyses in English. The words yes, no, mummy, daddy and their French equivalents, onomatopoeia which had no stable phonetic form, and interjections (e.g. ah, eh, oh) were excluded from the phonological sample. Calculations of PCC were computed automatically for each child based on the entire number of (non-code-switched) utterances in the language sample using the query function PCC in Phon. Phonological analyses in French were based on an average of 82 utterances (*SD* = 35; range = 13 – 147) and in English, on an average of 95 utterances (*SD* = 53; range = 13 – 212). Three participants were re-transcribed by a second transcriber using the Blind Transcription function of Phon. Point-to-point agreement in terms of consonant transcription was excellent in French (.97) and good in English (.86).

### Statistical analysis

The analyses included the following control variables: proportion exposure to English (Ex En), proportion exposure to French (Ex Fr), gender, age (in months) and maternal education (in years). The language variables were: percent consonants correct in English (En PCC), percent consonants correct in French (Fr PCC), raw vocabulary score in English based on the MCDI (En MCDI), NDW in English (En NDW), raw vocabulary score in French based on the French MCDI (Fr MCDI), number of different words in French (Fr NDW), MLU-words in English (En MLU), mean length of the three longest utterances in English based on parent report (En ML3), grammatical complexity in English based on parent report (En GramCom), MLU-words in French (Fr MLU), mean length of the three longest utterances in French based on parent report (Fr ML3), and grammatical complexity in French based on parent report (Fr GramCom).

Data were analysed using mixed-effect logistic regression, which allowed us to model production accuracy on the basis of binomial data. The analyses were performed using R statistical software (R Development Core Team, 2020) and the lme4 package (Bates et al., 2015) for mixed-effects models.

The dependent variable was PCC, which was coded as a proportion score (i.e. number of consonants correct/number of total consonants) for each individual word production. For example, voiture /vwatyʁ/ ‘car’ produced as [waty] was coded as 2/4. We also included a ‘weights’ argument in the model set to the number of total consonants to take into account that a proportion (e.g. 0.5) could refer to different numerators and denominators (e.g. 1/2, 2/4, 3/6, etc.). We conducted two separate statistical models: one to determine what factors influence English phonological performance and the other to determine what factors influence French phonological performance. To establish the most parsimonious model, we proceeded as follows. We first entered the control variables. We then entered variables for within-language vocabulary (MCDI and NDW) and grammatical measures (MLU) and finally we added variables for between-language phonology, vocabulary and grammatical measures. Due to the large number of language measures and the fact that ML3 and GramCom were subject to missing data, we did not include these variables in our models; however, we present these results in the descriptive statistics. At each step, we added all variables in one go and removed variables which were not significant. The final most optimal model was the one which had the lowest Akaike Information Criterion (AIC) and highest log likelihood ratio. The random part of the model included random intercepts for participants and items (i.e., words). The model was fitted using maximum likelihood estimation.

## Results

Table 2 presents descriptive statistics on the various language measures for the entire sample. In general, scores were slightly higher for French relative to English across all language measures (except GramCom) reflecting the fact that there were more children dominant in French than English. Nevertheless, there was a wide range of values for each language measure indicating that children had varied language proficiencies.

**Table 2.** Means and standard deviations (in parentheses) for phonological, vocabulary and grammatical measures for all children (*n* = 31).

	Mean (*SD*)	Range
Phonology		
En PCC^a^	81.24 (11.17)	48.28–93.99
Fr PCC	83.06 (5.85)	71.88–94.96
Vocabulary		
En MCDI	355.45 (187.91)	5–680
En NDW	89.19 (37.56)	20–149
Fr MCDI	374.90 (140.70)	56–590
Fr NDW	100.48 (33.27)	46–169
Grammar		
En MLU	2.09 (.52)	1.22–3.11
En ML3^b^	4.66 (2.45)	1–8.7
En GramCom^b^	16.4 (12.49)	0–37
Fr MLU	2.30 (.63)	1.42–3.45
Fr ML3^c^	5.22 (2.51)	1–10
Fr GramCom^b^	15.23 (11.50)	0–34

a En PCC: percent consonants correct in English; Fr PCC: percent consonants correct in French; En MCDI: raw vocabulary score in English based on the MacArthur Communicative Developmental Inventory (MCDI); En NDW: number of different words in English; Fr MCDI: raw vocabulary score in French based on the MCDI; Fr NDW: number of different words in French; En MLU: mean length of utterance-words in English; En ML3: mean length of the three longest utterances in English based on parent report; En GramCom: grammatical complexity in English based on parent report; Fr MLU: mean length of utterance-words in French; Fr ML3: mean length of the three longest utterances in French based on parent report; Fr GramCom: grammatical complexity in French based on parent report.

b Based on a total of 30 children: missing data on 1 child.

c Based on a total of 29 children; missing data on 2 children.

Table 3 presents the Pearson correlation coefficients between proportion of language exposure to English and English language measures and proportion of language exposure to French and French language measures. There were moderate positive correlations (ranging from .38 to .67) between language exposure and all language measures (phonology, vocabulary and grammar). Because we ran multiple tests, we used Benjamini-Hochberg corrections to yield adjusted significance levels (Benjamini & Hochberg, 1995). These corrections yielded varied effects of language exposure on the language measures (see Table 3). Language exposure had a significant influence on phonology measures in one of the two relations tested (i.e. Fr PCC); on vocabulary in three of the four relations tested (i.e., En MCDI, Fr MCDI, Fr NDW); and on grammar in four of the six relations tested (En MLU, En ML3, En GramCom, Fr ML3).

**Table 3.** Correlation coefficients between proportion exposure in English and English language measures and proportion exposure in French and French language measures.

English measures	Exposure to English	French measures	Exposure to French
Phonology			
En PCC	.33	Fr PCC	.47*^a^
Vocabulary			
En MCDI	.47*	Fr MCDI	.67**^b^
En NDW	.38	Fr NDW	.46*
Grammar			
En MLU	.59**	Fr MLU	.44
En ML3	.55*	Fr ML3	.52*
En GramCom	.51*	Fr GramCom	.43

Note: *p* values after Benjamini-Hochberg corrections have been applied.

a *p* < .05. ^b^**p < .01.

Table 4 presents the correlation coefficients between the language measures in English and French. Within-language correlations (shown in the upper left-hand quadrant for English and in the lower right-hand quadrant for French) were positive and ranged from .37 to .80 for English and .27 and .82 for French. Once again, we employed Benjamini Hochberg corrections to adjust for false positive error rate. Correlations which remained significant after corrections are highlighted in grey in the table. Results indicated that English phonology was correlated with English vocabulary and grammar (En NDW and En MLU), and English vocabulary was correlated with English grammar (En MCDI with En ML3 and En GramCom; EnNDW with En MLU and En ML3). French phonology was correlated with French vocabulary (Fr MCDI), and French vocabulary (Fr MCDI) was correlated with French Grammar (Fr MLU, Fr ML3 and Fr GramCom). There were also correlations between the grammar measures (En ML3 with En GramCom; FrMLU with Fr ML3 and Fr GramCom; Fr ML3 with Fr GramCom).

**Table 4.** Correlation matrix between phonology, vocabulary and grammar measures.

	En PCC	En MCDI	En NDW	En MLU	En ML3	En GramCom	Fr PCC	Fr MCDI	Fr NDW	Fr MLU	Fr ML3	Fr GramCom
**En PCC**	_	.38	.63**b	.63**	.53	.48	.06	−.25	−.23	−.21	−.10	−.13
**En MCDI**	_	_	.48	.41	.71***^c^	.80***	.01	−.09	−.39	−.22	−.25	−.19
**En NDW**	_	_	_	.74***	.62*^a^	.62	−.14	−.32	−.62*	−.44	−.26	−.44
**En MLU**	_	_	_	_	.53	.51	−.03	−.32	−.59*	−.24	−.16	−.15
**En ML3**	_	_	_	_	_	.73***	−.11	−.35	−.63*	−.34	−.08	−.16
**En GramCom**	_	_	_	_	_	_	.01	−.13	−.28	−.17	−.17	−.03
**Fr PCC**	_	_	_	_	_	_	_	.60*	.30	.48	.41	.49
**Fr MCDI**	_	_	_	_	_	_	_	_	.55	.65**	.66**	.72***
**Fr NDW**	_	_	_	_	_	_	_	_	_	.51	.27	.45
**Fr MLU**	_	_	_	_	_	_	_	_	_	_	.69**	.76***
**Fr ML3**	_	_	_	_	_	_	_	_	_	_	_	.82***
**Fr GramCom**	_	_	_	_	_	_	_	_	_	_	_	_

a *p < .05. ^b^**p < .01. ^c^***p < .001.

In addition, we observed significant between-language correlations; however, they were all negative. French NDW was negatively correlated with English NDW, English MLU and English ML3, meaning that saying more diverse words in a French session was associated with saying fewer diverse words in an English session and producing a smaller utterance length in English. In sum, zero-order correlations did not provide evidence for facilitative between-language effects. In particular, contrary to predictions, we did not observe positive cross-language correlations implicating phonology, vocabulary and grammar. However, these analyses do not control for the influence of language exposure, and other control variables. Hence, we explored predictive relations between phonological scores in one language and language measures in the same and in the other language using mixed logistic regression.

In the first model, we examined the factors that predict English phonological performance. As mentioned, the dependent variable was a proportion score (consonants correct/total number of consonants) for each individual English word spoken in the free-play session. There were 5701 individual items spoken by the children. In the first step, we examined the influence of control variables (age, proportion exposure, gender and maternal education) on English PCC. Results indicated that proportion exposure to English significantly influenced and gender marginally influenced English PCC. In the second step, we added within-language variables (i.e. En MCDI, En NDW, En MLU), while controlling for exposure and gender. Results indicated that both En MLU and En NDW were equal predictors of English phonological performance when added separately; so, in the third step, we examined whether between-language variables (Fr PCC, Fr MCDI, Fr NDW, Fr MLU) significantly contributed to English phonological performance when either En MLU or En NDW was included in the model. In the case of En MLU, no between-language variable significantly contributed to model fit, whereas in the case of En NDW, Fr NDW significantly contributed and Fr PCC marginally contributed to model fit. Based on the AIC score and log likelihood ratio, the optimal model contained proportion English exposure, En NDW, Fr NDW and Fr PCC. We retained the control variable gender in the final model as well. It is presented in Table 5.

**Table 5.** Optimal logistic regression model for predicting English phonology (percent consonants correct (PCC)).

Fixed effects	Estimate (β)	Standard error	z	*p v*alue
Intercept	2.237	0.155	14.394	< .001***c
ExEn	0.325	0.104	3.123	0.002**^b^
Gender	−0.225	0.188	−1.201	0.230
En NDW	0.325	0.091	3.554	< .001***
Fr NDW	0.21363	0.104	2.046	0.041*^a^
Fr PCC	0.19114	0.103	1.863	0.062
Random effects	Variance	*SD*		
Participant	0.096	0.310		
Word	1.062	1.030		

* *p* < .05. ^b^**p < .01. ^c^***p < .001.

In the second model, we examined the factors that predicted French phonological performance. The dependent variable was a proportion score (consonants correct/total number of consonants) for each French word spoken in the free-play session. There were 6333 individual items spoken by the children. In the first step, we examined whether control variables (age, proportion exposure, gender and maternal education) influenced French phonological performance. Results indicated that one control variable was significant; namely, proportion exposure to French. In the second step, we added within-language variables (Fr MCDI, Fr NDW, Fr MLU) while controlling for French exposure. Fr MLU was found to be the best predictor of French phonological performance. There was no other variable in combination with French MLU which emerged as significant. In the third step, we added between-language variables (En PCC, En MCDI, En NDW, En MLU) along with French MLU and proportion French exposure. We found that two variables, En PCC and En MLU, significantly contributed to model fit when added on their own. The AIC score and log likelihood ratios were very similar for both models with a slight preference for English MLU. Thus, we found that French phonological performance was best predicted by three factors: proportion French exposure, French MLU and English MLU, although an almost equally optimal model contained French exposure, French MLU and English PCC. The former model is presented in Table 6.

**Table 6.** Optimal logistic regression model for predicting French phonology (percent consonants correct (PCC)).

Fixed effects	Estimate (β)	Standard error	Z	*p* value
Intercept	2.086	0.081	25.652	<.001
ExFr	0.208	0.060	3.469	<.001***^b^
Fr MLU	0.186	0.055	3.384	<.001***
En MLU	0.181	0.061	2.974	0.003**^a^
Random effects	Variance	sd		
Participant	0.052	0.228		
Word	1.091	1.44		

a **p < .01. ^b^***p < .001.

In sum, statistical models provide some evidence of between-language effects. English phonological performance was best predicted by the number of different words in the same and in the other language. French phonological performance was best predicted by MLU scores in the same and in the other language. In addition, phonological scores in the other language were almost equally as effective as English MLU in predicting French phonology and were marginally significant on top of French NDW in predicting English phonology.

## Discussion

This study examined relations between phonology, vocabulary and grammar in French-English bilingual children. Previous studies focusing on the links between vocabulary and grammar in bilingual children have documented strong within- and weak between-language correlations (Conboy & Thal, 2006; Kohnert et al., 2010; Marchman et al., 2004). We posited that the relation between phonology and other language domains may be different from the one between vocabulary and grammar due to the language-general component of phonology reflecting speech motor ability as well as shared phonological knowledge between the two languages. Thus, we predicted, on the basis of correlational analyses, proportion language exposure would have a stronger influence on vocabulary and grammar than on phonology and that there would be between-language relations between phonology in one language and phonology, vocabulary and grammar in the other language. We also predicted, on the basis of mixed logistic regression, that phonological performance in one language would be influenced by the phonological, vocabulary and grammatical abilities of the other language. Our first prediction was not confirmed. In correlational analyses, there was a tendency for proportion exposure to be less strongly correlated with phonology than other language measures but the differences were very small. Furthermore, zero-order correlations revealed no significant cross-language correlations between phonology, vocabulary and grammar. Our second prediction was confirmed: in mixed-effect regression models, phonology in one language was related to language skills in the same and in the other language. In the following paragraphs, we discuss the results in more detail and compare them to previous studies which have examined cross-domain relations in bilingual children.

### Influence of language experience

Unsworth (2016) asked the question of whether input effects hold similarly across different language domains. She reviewed studies indicating that amount of input influences vocabulary and morphosyntactic acquisition but she did not include studies examining input effects on phonological acquisition. Our own literature review revealed that language exposure has a strong influence on lexical and morphosyntactic performance (Hoff et al., 2012; Legacy et al., 2016), but a less strong influence on phonological performance (Almeida et al., 2012; Goldstein et al., 2005, 2010; Meziane & MacLeod, 2017). We hypothesized that language exposure may exert a weaker influence on phonology than on other domains due to the language-general component of phonology which impacts both languages equally. In the area of vocabulary and morphosyntax, we obtained positive correlation coefficients ranging from .38 to .67 which are similar to those of Parra et al. (2011) when examining the relation between language exposure, vocabulary size and grammatical complexity in bilingual English-Spanish children, aged 25 months (.45 to .72). In the area of phonology, the magnitude of the correlation with language exposure (.33 to .47) was somewhat smaller than for vocabulary (.38 to .67) and grammar (.43 to .59). However, once we applied corrections to adjust for false positive error rate, not all language measures were correlated with language exposure. This was the case for phonology (En PCC), vocabulary (En NDW) and grammar (Fr MLU & Fr GramCom) alike. Furthermore, language exposure emerged as a significant predictor of phonological abilities in English and French in our statistical models, suggesting that quantity of input did indeed influence phonological skills at least for the bilingual children in the current dataset. In sum, there was not strong evidence that exposure influenced phonology less than other language domains.

### Relations between language domains

Our study focused on relations between language domains in bilingual children. We predicted between-language relations, either between the two phonological systems of the bilingual child (Cooperson et al., 2013; Keffala et al., 2020; Scarpino et al., 2019) or between the phonological system of one language and the lexical or grammatical system of the other (Cooperson et al., 2013; Kehoe & Havy, 2019).

Our correlational analyses did not support our predictions. The correlations between phonology and vocabulary, and phonology and grammar were akin to those obtained between vocabulary and grammar. They were predominantly within-language and of a similar magnitude. Between-language correlations, when present, were negative, meaning that high language skills in one area were associated with low language skills in another area. The majority of them involved the vocabulary measure, Fr NDW. High lexical diversity in French meant low lexical diversity and low grammatical scores in English. Since the negative correlations were largely confined to the behavioral task, we suspect they relate to specific aspects of the methodology. The caregiver who interacted with the child was a bilingual English-French parent who was often present for both the French and English sessions. Children may have developed language preferences for speaking with the parent in one language, leading to increased use of the favored language in one session to the detriment of the non-favored language in the other session. An alternative explanation may have its origin in socio-cultural influences and reflect the suppressing influence of language skills in one language on growth in the other (Hoff et al., 2018). Another possibility may relate to language typological differences. Subtractive between-language effects may emerge when languages differ significantly on phonological, lexical and grammatical dimensions, a finding to be confirmed in future studies.

The raw correlations did not control for language exposure (and other control variables), nor did they take into account random effects related to participants and items; thus, we entered the data into mixed-effect logistic regression models. The results revealed between-language effects for both English and French phonology. English phonology was influenced by vocabulary diversity (NDW) whereas French phonology was influenced by grammatical development (MLU) across the two languages of the bilinguals. Like Cooperson et al. (2013), we posit that language-general phonological skills may support the acquisition of language skills in both languages.

One might wonder why vocabulary was the best predictor of English phonology and grammar the best predictor of French phonology. We do not think this reflects any important differences in cross-domain relations between English and French phonology. In statistical models, same-language vocabulary and grammar measures were significant predictors of phonological skills when entered separately but not when entered together, probably due to the shared variance between language measures. We retained in the statistical models those variables which were the best predictors of phonological skills. In the case of English phonology, both NDW and MLU were significant predictors; however, between-language effects were only associated with the vocabulary variable, NDW. In the case of French phonology, the grammar variable, MLU, had a slight edge over the vocabulary variable, MCDI in the statistical model. It should be noted that Cooperson et al. (2013) observed stronger relations between phonology and grammar than between phonology and semantics in bilingual children. Others have reported strong positive relations between MLU scores and PCCs in bilingual children (Goldstein et al., 2010; Montanari et al., 2018). Our findings are consistent with their results (at least for French phonology) in indicating that vocabulary does not have a monopoly on phonology in cross-language relations. Studies which have examined phonological effects on grammatical morpheme acquisition may explain the strong connection we observed between phonology and utterance length in this study (Demuth & Tomas, 2016; Gerken, 1996; Lleó & Demuth, 1999).

One salient finding in the literature is that phonological skills in one language are associated with phonological skills in the other language (Cooperson et al., 2013; Keffala et al., 2020; Montanari et al., 2018; Scarpino et al., 2019). We also documented such between-language phonological influences in the current study although, in contrast to prior research with large samples, we did not observe them in the correlational analyses. In the regression analyses, in which we controlled for language exposure, gender, age and maternal education, phonology in one language predicted phonology in the other. However, phonology was not the strongest predictor of phonology in the other language when between-language vocabulary and grammar measures were entered into the statistical models. Previous studies have not necessarily included between-language measures of vocabulary and grammar along with phonology. An exception is Cooperson et al. (2013) who found that variance in English and Spanish phonology was best accounted for by within- and between-language grammatical measures; in their case, between-language phonological measures were not retained in the statistical models. Please note that we cannot conclude that the relation of phonology to other language domains is only language general. Given the fact that cross-language variables were not the strongest predictors in the regression models, there is also evidence for language specificity. It would be interesting to examine the extent to which the phonological proximity of the bilingual’s two languages (e.g. overlap in phonetic and syllable structure inventories, rhythm differences) influences the magnitude of language general versus language specific cross-language effects.

In terms of methodology, our study differs from previous studies which have examined cross-domain relations in bilingual children. We investigated French-English bilinguals whereas all the studies we are aware of have tested Spanish-English bilinguals. We tested young children, aged on average 31 months, whereas those studies, which have reported between-language relations in phonology, have all tested children aged 3 years and older. We employed a spontaneous language sample to extract measures of phonological ability, whereas other studies have employed single-word production tasks. Given these differences in methodology, as well as the fact that we tested fewer participants than the large-scale studies of Cooperson et al. (2013), Keffala et al. (2020) and Scarpino et al. (2019), it is striking that we still obtained relatively similar results. It could be posited that French and Spanish, both being Romance languages, and both being characterized by syllable-timed rhythm and a high predominance of multisyllabic words, may have similar shared phonological characteristics with that of English. Thus, commonalities in the linguistic properties of French and Spanish may lead to similar cross-domain effects in the two bilingual language groups.

In terms of age, the inclusion of younger children in the current study could have meant greater variability of phonological scores in both languages, reducing the chances of observing significant between-language correlations in phonology. Instead, we documented significant effects (at least in the regression analyses), which, in combination with the between-language effects reported in children aged three to six years, seem to suggest continuity over time in between-language cross-domain relations. A closer examination of the magnitude of these effects and how they change over time would need to be conducted, however, with a longitudinal research design.

Finally, the use of a different sampling condition (conversational sample vs single-word production test) could have potentially led to some differences between ours and previous studies. Morrison and Shriberg (1992) observed that the contribution of cognitive-linguistic and pragmatic processes are different in the two sampling modes. On the one hand, the act of formulating sentences from thought in a conversational situation results in a more cognitively demanding task; on the other hand, the liberty of choosing words and sentence structures within one’s production capacities leads to a less demanding task. It is possible that the use of the spontaneous speech sample to collect phonological measures may have rendered the measures more ‘language-like’, and, thus, we did not observe significant zero-order correlations between the two phonology measures as has been reported by other investigators. Only in more sophisticated statistical modeling did the between-language effects emerge. Future studies should examine what type of sampling mode is the most effective means for examining cross-domain relations in phonology.

Our findings support our hypothesis in revealing between-language relations between phonology and other language domains. Nevertheless, as noted in the introduction, a handful of studies have reported between-language effects in vocabulary and grammar (Conboy & Thal, 2006; Dixon, 2011; Kohnert et al., 2010; Marchman et al., 2004; Pham, 2016). Therefore, to provide stronger support for our hypothesis, we would need to conduct a study in which vocabulary and grammatical measures were subject to the same statistical modeling as phonology was in the current study. Furthermore, we have tested cross-domain relations using phonology as the dependent variable, and it would be interesting to examine the strength of between-language relations when vocabulary and grammar are employed as dependent variables. These studies remain to be conducted.

## Conclusion

At the outset of this study, we hypothesized that relations between phonology and other language domains in bilingual children operate differently from those between vocabulary and grammar (Conboy & Thal, 2006; Marchman et al., 2004). Phonological skills of bilinguals may be supported by a language-general articulatory component and by language-specific phonological components which resemble language-general-like components due to the strong resemblances which exist between phonetic inventories and phonotactic structures across languages. Our findings have clinical implications for language remediation in bilingual children since they reveal that strong phonology in one language has the potential to bootstrap phonology, vocabulary and grammar in the other language (French phonology contributed to the model predicting English phonology and English phonology was a close competitor to English MLU in predicting French phonology). It would be important to determine whether such effects exist across all populations of bilingual children, or only those growing up in additive bilingual environments such as the French-English bilinguals in this study. Given that facilitative between-language effects have also been reported in Spanish-English bilinguals in the US (Cooperson et al., 2013; Keffala et al., 2020), bilinguals who are not necessarily growing up in additive contexts, these findings may be generalizable to other bilingual contexts. Finally, our study has focused on a single age-group and it would be important to examine between-language effects in a longitudinal research design to understand the dynamic interplay that exists between language domains over time.

## Notes

1. The BESA was in preparation at the time Cooperson et al. (2013) were conducting their study. It has subsequently been published (Peña et al., 2018).

2. Paradis and Kirova (2014) report that bilingual profile effects may be evident when examining children’s narrative skills with greater monolingual-bilingual differences present in vocabulary and morphosyntactic measures which reflect distributed knowledge and fewer differences present in story grammar which reflects shared knowledge. Thus, shared knowledge may be evident in other language domains apart from phonology.

3. We included utterances in which only one word was code-switched within the utterance so as to avoid excluding too many utterances.

4. The language samples were transcribed by European French-speaking students whereas the children spoke Canadian French. Before transcription, the students received information on differences between Canadian and European French. They participated in training sessions in which these differences were discussed. Good inter-rater reliability (> .85) was obtained among the 3 students before they started transcription.
